# Analysis of the interhospital transfer times in patients with ST-elevation acute coronary syndrome (STEMI) for undergoing urgent coronariography

**DOI:** 10.1186/cc14243

**Published:** 2015-03-16

**Authors:** FM Acosta Diaz, O Moreno, M Muñoz, R Fernandez, J Soto, M Colmenero

**Affiliations:** 1 San Cecilio Universitary Hospital, Granada, Spain

## Introduction

To analyze the related assistance times to transfer patients with STEMI referred to another hospital with a hemodynamics unit (HU) for performing emergency catheterization (primary or rescue PCI).

## Methods

A consecutive registry of patients seen in 2013 (January to October) in the ICU of a hospital without a HU. The total transfer time is considered from the call to the Emergency Coordination Center until arrival at the HU. In turn, this time is divided into activation time, arrival time of the relocation team, patient preparation time and transfer time. In the case of primary PCI, the door-to-balloon time was estimated by adding to the total transfer time the initial assessment and completion time of catheterization and balloon inflation. The times are expressed in minutes, as the median and interquartile range.

## Results

During 10 months of 2013, we treated 162 STEMI. Of these, 104 had evidence of reperfusion (64%). Primary PCI was performed in 24 patients (23%), of which 10 were transferred from the hospital to the HU. Fibrinolytic therapy was used in 62 patients (59%), of these 20 (32.2%) required rescue PCI. The transfer time for primary PCI was 0:39:44 (0:31:41 to 0:44:32) minutes. The transfer time for rescue PCI was 0:38:56 (0:37:25 to 0:51:29) minutes. The door-to-balloon time estimated for primary PCI was 80 minutes.

## Conclusion

Times for interhospital transfer of patients with STEMI who had undergone urgent catheterization are within the range considered optimal. In the case of primary PCI, times are lower than the 90 to 120 minutes recommended practice guidelines.

